# A novel prediction model for the completion of six cycles of radium-223 treatment and survival in patients with metastatic castration-resistant prostate cancer

**DOI:** 10.1007/s00345-021-03639-z

**Published:** 2021-03-01

**Authors:** Yasuhide Miyoshi, Sohgo Tsutsumi, Masato Yasui, Takashi Kawahara, Ko-ichi Uemura, Naruhiko Hayashi, Masahiro Nozawa, Kazuhiro Yoshimura, Hiroji Uemura, Hirotsugu Uemura

**Affiliations:** 1 Department of Urology and Renal Transplantation, Yokohama City University Medical Center, 4-57 Ufafune-cho Minami-ku, Yokohama, Kanagawa 232-0024 Japan; 2 Department of Urology, Yokohama City University Graduate School of Medicine, 3-9 Fukuura Kanazawa-ku, Yokohama, Kanagawa 236-0004 Japan; 3 Department of Urology, Kindai University Faculty of Medicine, 377-2 Ohno-higashi, Osakasayama, Osaka 589-8511 Japan

**Keywords:** Prostate cancer, Radium-223, Hemoglobin, Alkaline phosphatase, Pain

## Abstract

**Purpose:**

We evaluated the predictive factors for completion of all six cycles of radium-223 (Ra-223) treatment in patients with metastatic castration-resistant prostate cancer (mCRPC). We also developed a novel prediction model for Ra-223 treatment completion using these predictors.

**Methods:**

We retrospectively reviewed data from 122 patients with mCRPC who were treated with Ra-223. The predictive factors for the completion of six cycles of Ra-223 treatment were evaluated. Statistically significant predictive factors were then used to develop a prediction model for treatment completion. Finally, using this prediction model, we classified the overall survival (OS) of the entire cohort into three groups.

**Results:**

We identified three significant variables as the predictive factors for treatment completion: baseline alkaline phosphatase (ALP) level, baseline hemoglobin (Hb) level, and baseline pain. The three groups generated using the prediction model were: group 1 (patients with three predictive factors, i.e., ALP < median, Hb ≥ median, and no pain), group 2 (patients with one to two predictive factors), and group 3 (patients without any predictive factors). The treatment completion rates differed between the three groups significantly. Furthermore, the OS also differed among the groups significantly.

**Conclusion:**

Our study suggested that the baseline ALP level, baseline Hb level, and baseline pain were the predictive factors of completion of all six cycles of Ra-223 treatment in patients with mCRPC. Our prediction model consisting of these factors could predict not only the completion of Ra-223 treatment, but also the post-treatment survival. This model can thus be useful for selection of patients for Ra-223 treatment.

**Supplementary Information:**

The online version contains supplementary material available at 10.1007/s00345-021-03639-z.

## Introduction

Radium-223 (Ra-223) is an alpha-emitting radionuclide that selectively targets bone metastases [1]. The Alpharadin in Symptomatic Prostate Cancer Patients (ALSYMPCA) study (a phase III, randomized, double-blind, and multinational study) demonstrated that Ra-223 treatment led to an improvement in not only the survival, but also the health-related quality of life of patients with metastatic castration-resistant prostate cancer (mCRPC) [1–3]. In this study, the median overall survival (OS) of patients treated with Ra-223 and the placebo was 14.0 and 11.2 months, respectively, and the hazard ratio (HR) was 0.70 (95% confidence interval [CI] 0.55–0.88).

Based on these findings, Ra-223 was established as a standard treatment modality for mCRPC patients with predominant bone disease and no visceral metastases [4–6]. The approved regimen of Ra-223 is an intravenous injection of 55 kBq/kg of Ra-223, every 4 weeks (q4w), for six cycles [1, 4].

The biological effectiveness of a radionuclide therapy is dose-dependent [7]. Saad et al. conducted a phase IIIb, single-arm study in the setting of an international early access program (iEAP) [8] that evaluated the correlation between the number of treatment cycles of Ra-223 (1–4 vs. 5–6) and the OS using a post hoc analysis [7]. The primary endpoints of this study were the safety and survival of patients treated with Ra-223 for symptomatic or asymptomatic mCRPC. It was found that patients who completed the treatment achieved a better clinical outcome (5–6 cycles; median OS: not reached) than those who discontinued early (1–4 cycles; median OS: 6.3 months). Therefore, completion of Ra-223 treatment is necessary for improving survival. However, the predictors for Ra-223 treatment completion have not been established yet. Therefore, in this study, we evaluated the predictors for completion of six cycles of Ra-223 treatment in mCRPC patients and also developed a novel prediction model for the same.

## Patients and methods

### Patients

We retrospectively reviewed 122 patients with bone-mCRPC who were treated with Ra-223 between 2012 and 2020 at the Yokohama City University Medical Center and the Kindai University Hospital. Some patients had participated in the Japanese trial on Ra-223. All patients presented with histologically confirmed conventional prostate adenocarcinoma. Patients with visceral metastasis and/or regional lymph node metastases measuring greater than 4 cm were excluded. Patients were administered with Ra-223 intravenously at a dose of 55 kBq/kg, q4w, for up to six cycles. Additionally, all patients were also administered with a luteinizing hormone-releasing hormone (LHRH) agonist or antagonist. In some patients, abiraterone acetate (ABI) was administered simultaneously with Ra-223 before the results of the phase III ERA-223 trial were obtained; this trial demonstrated that compared to ABI alone, the combination of Ra-223 and ABI increased the incidence of bone fracture [9]. Furthermore, bone-modifying agents (BMAs) such as zoledronic acid (4 mg) or denosumab (120 mg) were also administered (qw4) in accordance with the physicians’ judgement.

Prostate-specific antigen (PSA) levels and laboratory data were evaluated q4w, and computed tomography and/or bone scanning with 99 m-technetium were performed every 12 weeks. In cases of cancer progression or severe adverse events, the physicians ceased the Ra-223 treatment before six cycles were completed and changed it.

After Ra-223 failure, all patients continued to receive LHRH agonist or antagonist, and were subsequently treated with ABI, enzalutamide (ENZ), docetaxel, cabazitaxel, steroids, and/or the best supportive care, in accordance with each physician’s treatment strategy.

All experimental procedures were conducted in accordance with the ethical standards of the Helsinki Declaration. This study was approved by the institutional review board of the Yokohama City University Medical Center (B181000040). Informed consent for participation in this study was obtained from all subjects in an opt-out manner.

### Clinical assessments

The following data were collected from electronic medical records: the patient’s age; concurrent use of ABI, ENZ, and BMA; baseline alkaline phosphatase (ALP), hemoglobin (Hb), lactate dehydrogenase (LDH), and PSA levels; baseline pain; history of docetaxel use; number of Ra-223 treatment cycles; and survival. Patients without pain were included in this study, because Ra-223 was approved for both, mCRPC patients with and without pain in Japan. CRPC was defined according to the Prostate Cancer Working Group 2 criteria [10]. Pain was defined by the patient’s subjective judgement.

### Statistical analysis

All analyses were conducted using IBM SPSS Statistics software for Windows, version 26 (IBM Corp., Armonk, NY, USA) and EZR (Saitama Medical Center, Jichi Medical University, Saitama, Japan) [11]. Data on continuous variables were divided into binary variables based on the median value of each variable.

The Fisher’s exact test was to evaluate the distribution of the frequency of each variable in different groups, namely, patients with treatment completion and patients without treatment completion). The Mann–Whitney *U* test was used to compare the differences in the running variables between those two groups.

The primary endpoints of this study were the predictive factors of completion of six cycles of Ra-223 treatment. Multivariate analysis using a logistic regression model was used to extract significant predictive factors for completion of six cycles of Ra-223 treatment, and the OR and 95% CI for each variable were determined. On the basis of statistically significant predictive factors, we developed a prediction model for treatment completion; this model was used to classify the entire cohort into three groups.

The Kaplan–Meier product-limit estimator was used to assess the OS distribution, and the log-rank test was used to analyze the differences in the OS between the groups. All tests were two-sided, and *p* < 0.0.5 was considered as statistically significant.

## Results

The patients’ characteristics are listed in Table 1. The median observation time was 11.0 months (range 0.6–56.5 months).

**Table 1:** Patient characteristics

	Entire cohort (*n* = 122)	Patients with treatment completion (*n* = 83)	Patients without treatment completion (*n* = 39)	*p* value*
Median age (range), years	75 (49–92)	76 (57–92)	74 (49–90)	0.273
Concomitant use of ARTA, *n*, %	40 (32.8)	34 (41.0)	6 (15.4)	0.007
Median baseline ALP level (range), IU/L	328 (113–3,540)	275 (113–2,584)	562 (163–3540)	< 0.001
Median baseline Hb level (range), g/dL	12.3 (7.5–15.5)	12.6 (7.5–15.5)	10.8 (8.0–15.2)	< 0.001
Median baseline LDH level (range), IU/L	213 (110–528)	208 (111–512)	221 (110–528)	0.366
Median baseline PSA level (range), ng/mL	35.9 (0.0–3157.0)	23.1 (0.0–859.0)	79.3 (2.9–3157.0)	< 0.001
Concomitant use of BMA; *n* (%)	46 (37.7)	32 (38.6)	14 (35.9)	0.843
Baseline pain; *n* (%)	52 (42.6)	29 (34.9)	23 (59.0)	0.018
Previous use of docetaxel, *n* (%)	42 (34.4)	27 (32.5)	15 (38.5)	0.545

*ARTA* androgen receptor-targeted agent, *ALP* alkaline phosphatase, *Hb* hemoglobin, *LDH* lactate dehydrogenase, *PSA* prostate-specific antigen, *BMA* bone-modifying agent

*The difference between patients with treatment completion and those without treatment completion

Of the 122 patients analyzed, 83 patients (68.0%) completed all six cycles of Ra-223 treatment. Of the remaining 39 patients, 8 (6.6%), 5 (4.1%), 5 (4.1%), 9 (7.4%), and 12 (9.8%) patients completed one, two, three, four, and five cycles, respectively. The reasons for completing five or less cycles of treatment were cancer progression in 30 patients (PSA progression, worsening of general health condition, bone metastasis progression, increased pain, new visceral metastasis in the liver, new visceral metastasis in the lung, and cancer-related death in 6, 13, 6, 2, 1, 1, and 1 patients, respectively), medical events (except prostate cancer progression) in 3 patients (cerebrovascular events, arrhythmia, and anemia in one patient each), patient’s desire in 2 patients, and unknown causes in 2 patients.

We identified three variables as the significant predictive factors for the completion of Ra-223 treatment, namely the baseline ALP level, baseline Hb level, and baseline pain (Table 2). Furthermore, we also developed a prediction model for treatment completion consisting of these three variables. This model could classify the entire cohort into three groups: group 1 involved patients with all three predictive factors (i.e., patients with ALP < median, Hb ≥ median, and no pain) (*n* = 23); group 2, patients with one to two predictive factors (*n* = 81); and group 3, patients without any predictive factors (*n* = 18).

**Table 2:** Univariate and multivariate analyses for predictive factors of completion of six cycles of radium-223 treatment

	Univariate analysis				Multivariate analysis			
	OR	95% CI		*p* value	OR	95% CI		*p* value
		Lower	Upper			Lower	Upper	
Concomitant use of ARTA (yes vs. no)	0.262	0.099	0.694	0.007	0.338	0.112	1.015	0.053
Median baseline ALP levels (> 328 IU/L vs. ≤ 328 IU/L)	4.057	1.777	9.264	0.001	3.225	1.117	9.311	0.030
Median baseline Hb levels (> 12.3 g/dL vs. ≤ 12.3 g/dL)	5.889	2.469	14.044	< 0.001	7.156	2.407	21.274	< 0.001
Median baseline LDH levels (> 285 IU/L vs ≤ 285 IU/L)	1.533	0.712	3.297	0.275	1.121	0.398	3.161	0.829
Median baseline PSA levels (> 35.9 ng/mL vs ≤ 35.9 ng/mL)	3.243	1.445	7.279	0.004	2.604	0.939	7.219	0.066
Concomitant use of BMA (yes vs. no)	0.893	0.405	1.966	0.778	0.355	0.115	1.098	0.072
Baseline pain (yes vs. no)	2.677	1.225	5.848	0.014	3.004	1.112	8.116	0.030
Previous use of docetaxel (yes vs. no)	1.296	0.587	2.862	0.521	0.520	0.175	1.546	0.240

*ARTA* androgen receptor-targeted agent, *ALP* alkaline phosphatase, *Hb* hemoglobin, *LDH* lactate dehydrogenase, *PSA* prostate-specific antigen, *BMA* bone-modifying agent, *OR* odds ratio, *CI* confidence interval

According to our prediction model, the completion rates of Ra-223 treatment in groups 1, 2, and 3 were 100% (23/23), 67.9% (55/81), and 27.8% (5/18), respectively. There were statistically significant differences in the completion rates between each group (group 1 vs. group 2: *p* < 0.001, group 1 vs. group 3: *p* < 0.001, and group 2 vs. group 3: *p* = 0.002).

**Fig. 1:**
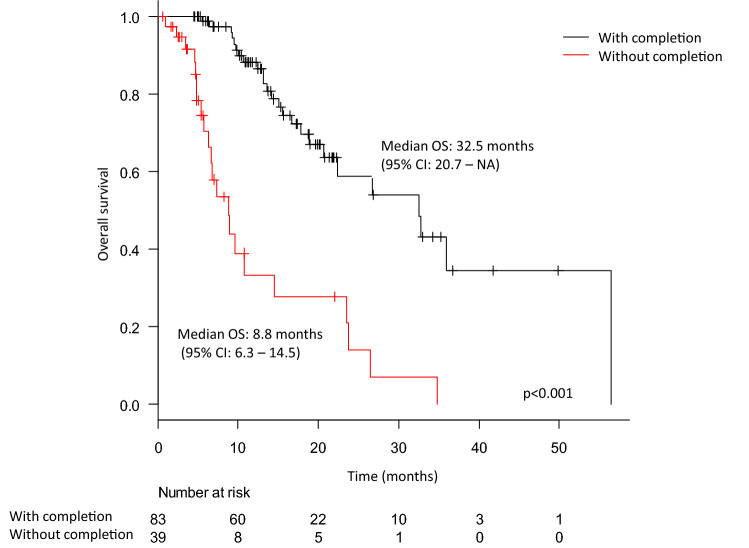
The Kaplan–Meier curves for overall survival according to Ra-223 treatment completion. The black and red lines indicate the survival of patients with and without completion, respectively

The median survival of the entire cohort was 23.5 months (95% CI 16.7–32.5 months) (Supplementary Fig. 1). The Kaplan–Meier curves according to treatment completion are shown in Fig. 1. The median OS differed significantly between patients who completed and did not complete the treatment and was 32.5 months and 8.8 months, respectively, (*p* < 0.001).

The Kaplan–Meier curves according to the baseline ALP level, baseline Hb level, and baseline pain are shown in Supplementary Figs. 2, 3, and 4, respectively. The median OS of the patients with ALP < median, ALP > median, Hb ≥ median, Hb < median, without pain, and with pain was 32.8 months (95% CI 22.4–not available [NA]), 15.5 months (95% CI 10.6–23.7 months), 32.5 months (95% CI 18.9–NA), 15.5 months (95% CI 10.6–23.7 months), 34.8 months (95% CI 23.5–NA), and 15.1 months (95% CI 10.1–22.4 months), respectively.

Patients who presented with ALP < median, Hb ≥ median, and no pain had a significantly longer survival than those who did not.

**Fig. 2:**
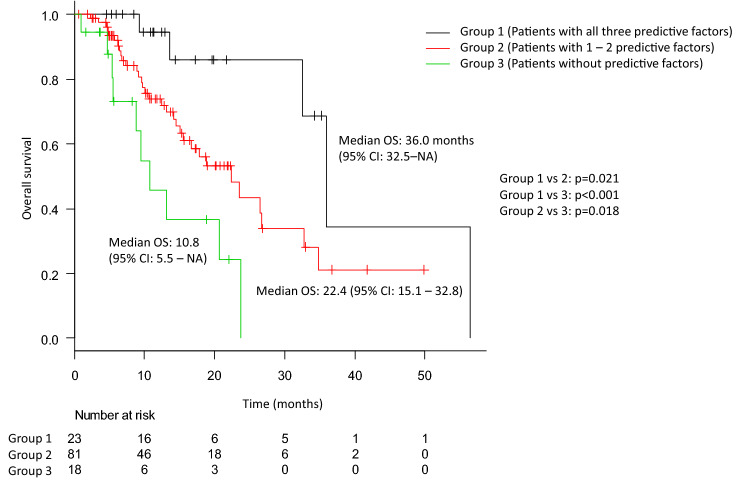
The Kaplan–Meier curves for overall survival according to our predictive model consisting of the baseline alkaline phosphatase (ALP) levels, hemoglobin (Hb) levels, and pain. The black, red, and green lines indicate the survival of patients in group 1, patients with all three predictive factors (i.e., patients with ALP < median, Hb ≥ median, and no pain), group 2, patients with one to two predictive factors, and group 3, patients without any predictive factors, respectively

Finally, the Kaplan–Meier curves according to our prediction model are shown in Fig. 2. According to this model, the median OS of the patients in groups 1, 2, and 3 was 36.0 months (95% CI 32.5–NA), 22.4 months (95% CI 15.1–32.8 months), and 10.8 months (95% CI 5.5–NA), respectively. There were significant differences in the OS between all groups (group 1 vs. group 2: *p* = 0.021, group 1 vs. group 3: *p* < 0.001, and group 2 vs. group 3: *p* = 0.018).

## Discussions

To improve the oncological outcomes after Ra-223 treatment, the completion of all six cycles of treatment is required [7, 12]. Therefore, it is necessary to identify the predictive factors for the completion of Ra-223 treatment. Saad et al. reported that patients treated with 5–6 cycles of Ra-223 treatment had a longer OS than those treated with 1–4 cycles (median OS: not reached vs. 6.3 months). Furthermore, less pain (non-mild), a good Eastern Cooperative Oncology Group performance status (0–1), lower PSA level (≤ 141 ng/mL), and higher Hb (≥ 10 g/dL) were the predictive factors for completing five to six cycles of treatment [7]. In a population-based study, Parimi et al. also reported that patients in the ≥ five cycles group had a longer OS as compared to those in the < five cycles group (median OS 16.2 vs. 5.9 months). They reported that a lower ALP level (< 220/IU/L) and higher Hb level (> 11.8 g/dL) were the predictive factors for completing from five to six cycles of treatment [12]. Our retrospective study also demonstrated that patients treated with six cycles of treatment had a significantly longer OS as compared to those treated with five or fewer cycles of treatment. Moreover, multivariate analysis demonstrated that ALP level < median, Hb level ≥ median, and no pain were the significant predictive factors for Ra-223 treatment completion. Based on these results, we developed a predictive model for the completion of Ra-223 treatment consisting of ALP, Hb, and pain. Our model could stratify the entire cohort into three groups according to the number of predictive factors. It could predict not only the completion of Ra-223 treatment, but also the survival after Ra-223 treatment.

This study had some limitations. Ra-223 is only approved for symptomatic CRPC patients by the US Food and Drug administration [13] and European Medicines Agency [14]. Therefore, the applicability of our model in patients with asymptomatic CRPC in the US and European countries remains unclear. However, despite the retrospective nature of our study and the small number of patients, our findings indicate that the Hb and ALP levels should be considered when the physician decides upon the use of Ra-223 in patients with mCRPC. For the confirmation of our results, a prospective study is warranted in the future.

## Conclusions

Our retrospective study suggested that the baseline ALP level, baseline Hb level, and baseline pain were the predictive factors for completion of six cycles of Ra-223 treatment in patients with mCRPC. Our prediction model consisting of these three predictors could predict not only the completion of six cycles of Ra-223, but also the survival post-treatment. Therefore, our prediction model could be useful for selecting patients for Ra-223 treatment.

## Supplementary Information

Below is the link to the electronic supplementary material.

**Supplementary Fig. 1** The Kaplan–Meier curves for the overall survival of the entire cohort. **Supplementary Fig. 2** The Kaplan–Meier curves for the overall survival according to the baseline alkaline phosphatase (ALP) levels. The black and red lines indicate the survival of patients with ALP ≤median and ALP>median, respectively. **Supplementary Fig. 3** The Kaplan–Meier curves for the overall survival according to the baseline hemoglobin (Hb) levels. The black and red lines indicate the survival of patients with Hb> median and Hb ≤median, respectively. **Supplementary Fig. 4** The Kaplan–Meier curves for the overall survival according to the baseline pain. The black and red lines indicate the survival of patients without and with pain, respectively. (PPTX 65 KB)

## Data Availability

The datasets generated during and/or analysed during the current study are available from the corresponding author on reasonable request.
