# Supplementary material for: A novel prediction model for the completion of six cycles of radium-223 treatment and survival in patients with metastatic castration-resistant prostate cancer
Source: World J Urol. 2021 Mar 1;39(9):3323–8. doi: 10.1007/s00345-021-03639-z (PMC8510910; doi:10.1007/s00345-021-03639-z)
Supplement: Supplementary file 1 — Supplementary Fig. 1 The Kaplan–Meier curves for the overall survival of the entire cohort. Supplementary Fig. 2 The Kaplan–Meier curves for the overall survival according to the baseline alkaline phosphatase (ALP) levels. The black and red lines indicate the survival of patients with ALP ≤median and ALP>median, respectively. Supplementary Fig. 3 The Kaplan–Meier curves for the overall survival according to the baseline hemoglobin (Hb) levels. The black and red lines indicate the survival of patients with Hb> median and Hb ≤median, respectively. Supplementary Fig. 4 The Kaplan–Meier curves for the overall survival according to the baseline pain. The black and red lines indicate the survival of patients without and with pain, respectively. (PPTX 65 KB) [file 345_2021_3639_MOESM1_ESM.pptx]

## Slide 1
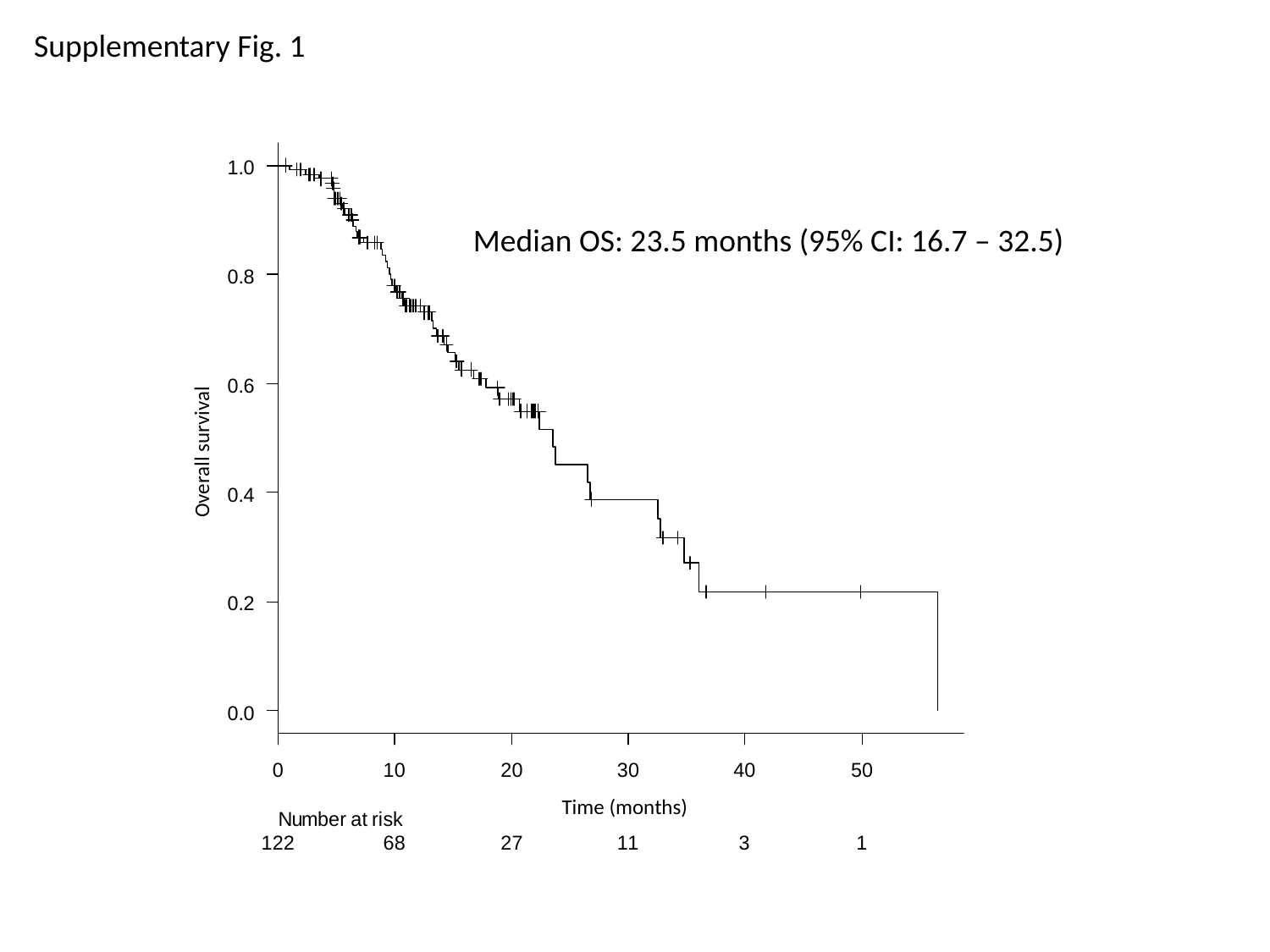

Supplementary Fig. 1
Median OS: 23.5 months (95% CI: 16.7 – 32.5)
Overall survival
Time (months)

## Slide 2
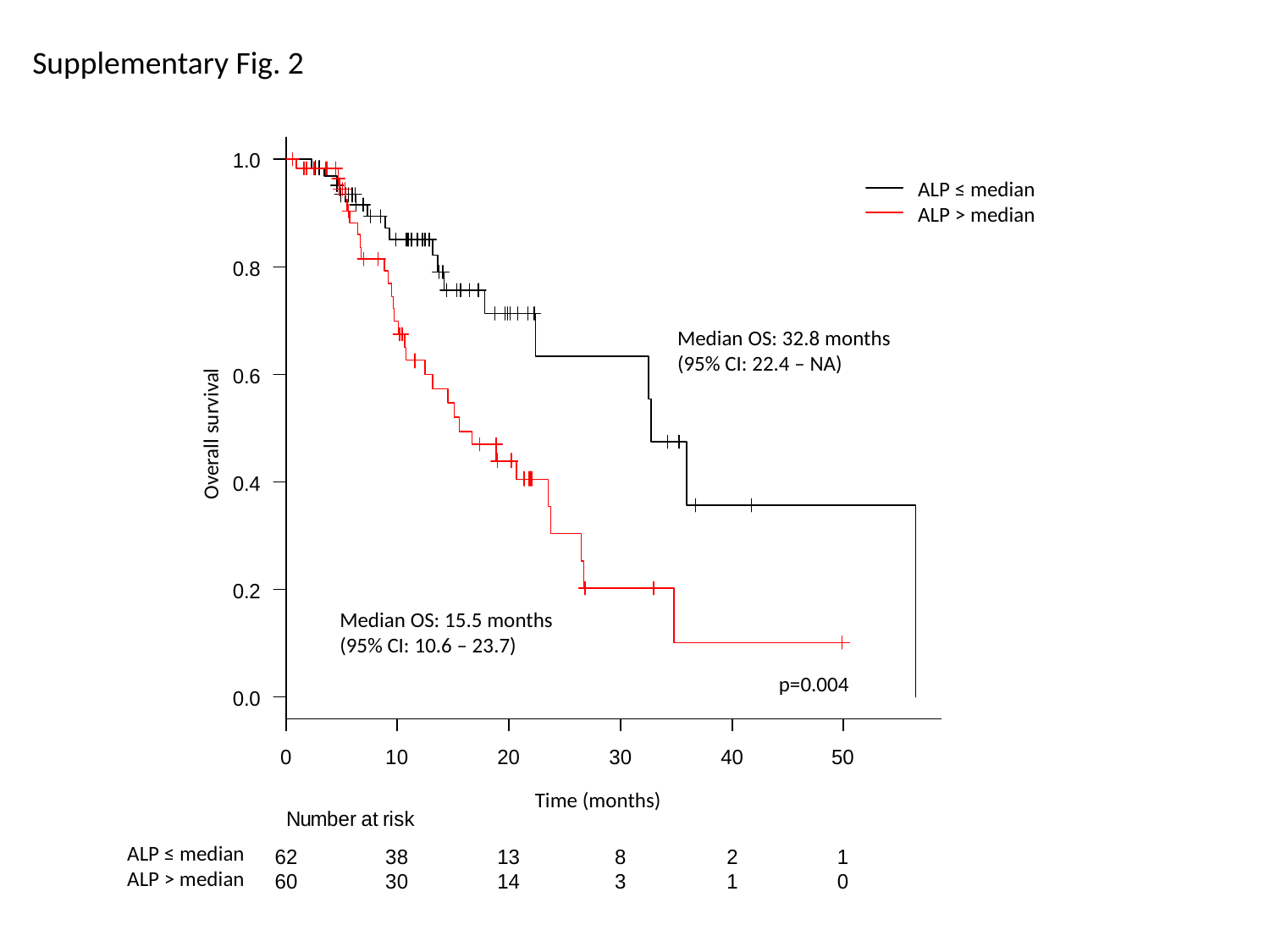

Supplementary Fig. 2
ALP ≤ median
ALP > median
Median OS: 32.8 months
(95% CI: 22.4 – NA)
Overall survival
Median OS: 15.5 months
(95% CI: 10.6 – 23.7)
p=0.004
Time (months)
ALP ≤ median
ALP > median

## Slide 3
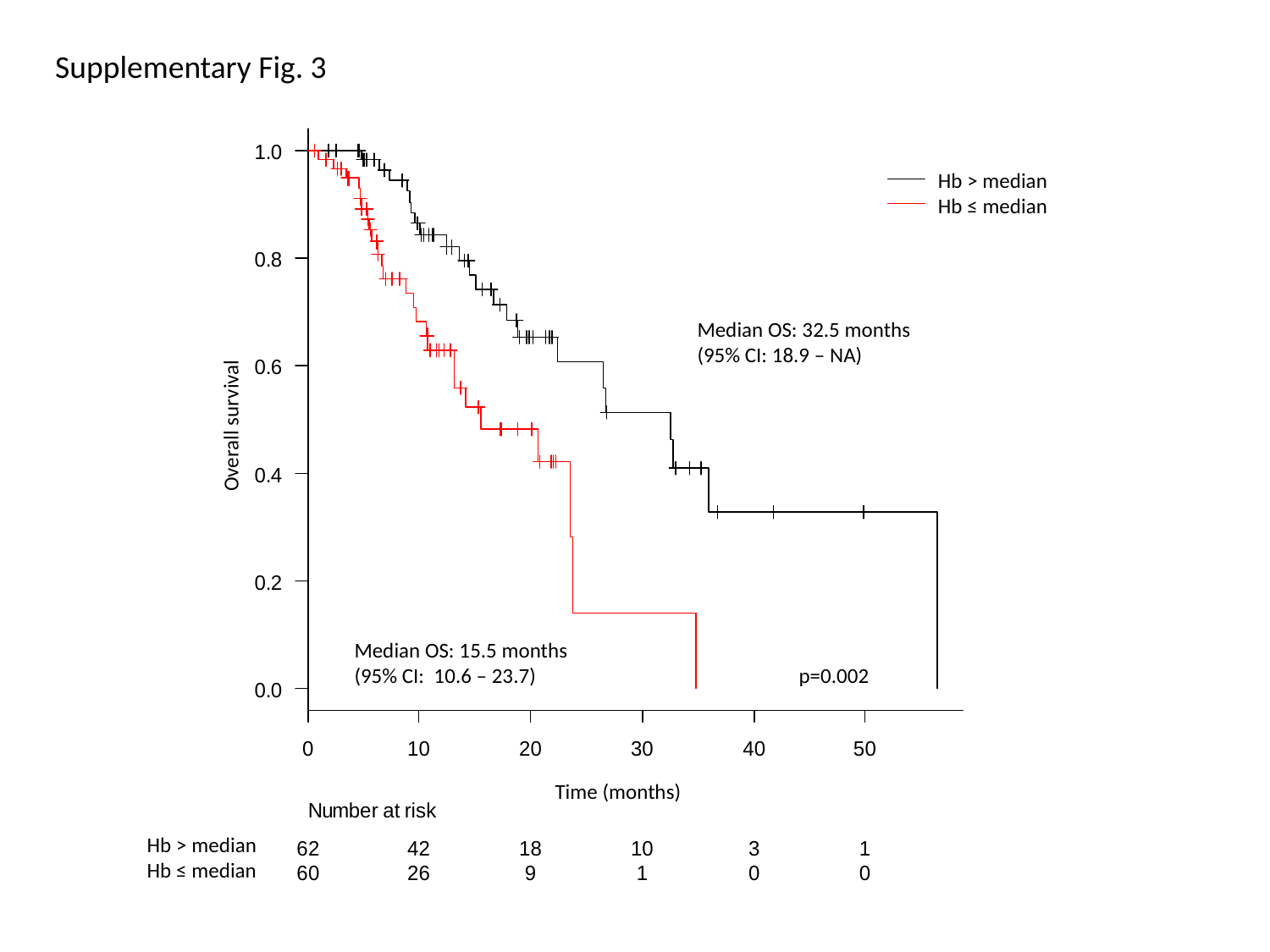

Supplementary Fig. 3
Hb > median
Hb ≤ median
Median OS: 32.5 months
(95% CI: 18.9 – NA)
Overall survival
Median OS: 15.5 months
(95% CI: 10.6 – 23.7)
p=0.002
Time (months)
Hb > median
Hb ≤ median

## Slide 4
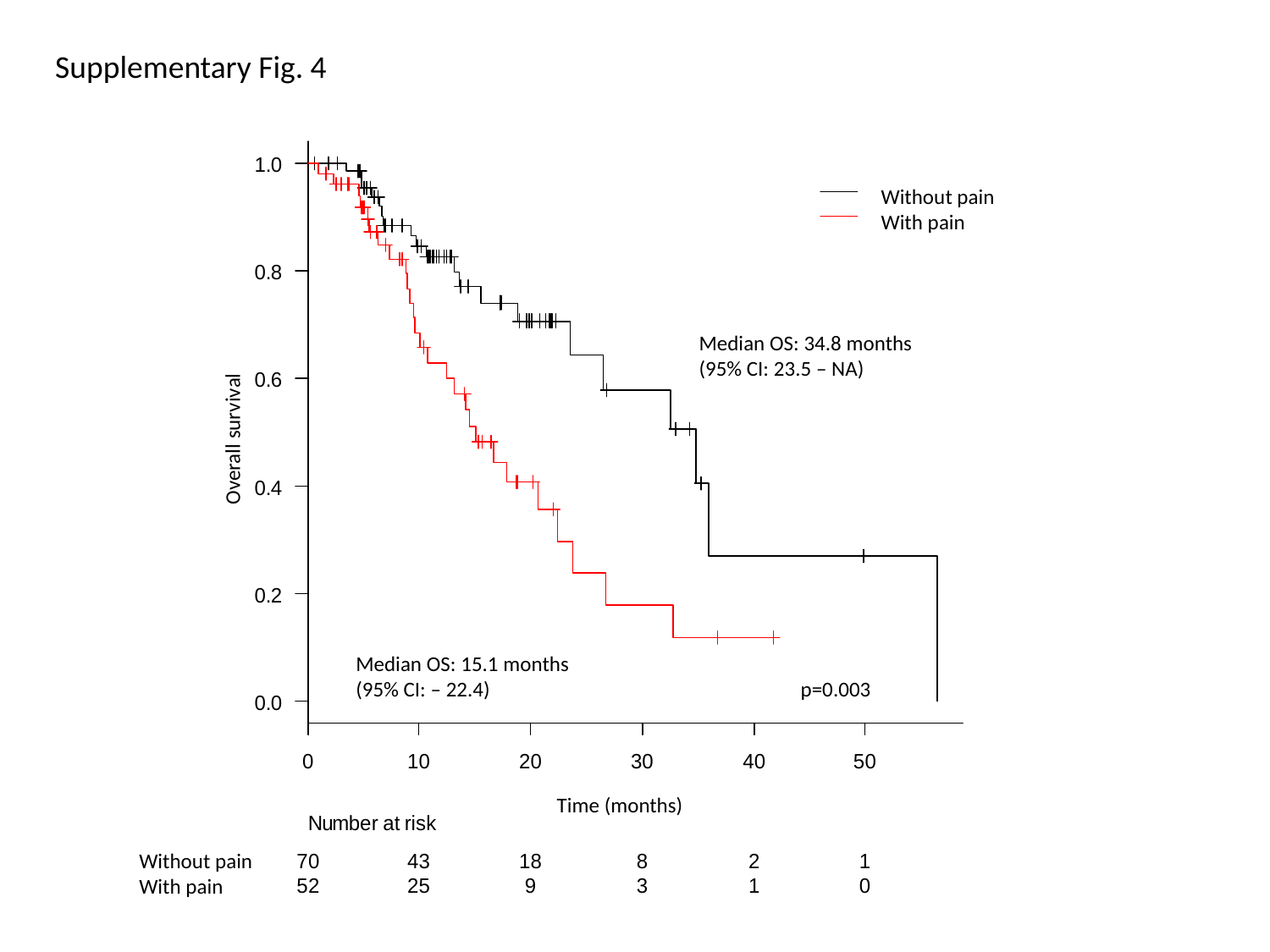

Supplementary Fig. 4
Without pain
With pain
Median OS: 34.8 months
(95% CI: 23.5 – NA)
Overall survival
Median OS: 15.1 months
(95% CI: – 22.4)
p=0.003
Time (months)
Without pain
With pain
